# Relationship between Athletes’ Big Five Model of Personality and Athletic Performance: Meta-Analysis

**DOI:** 10.3390/bs14010071

**Published:** 2024-01-19

**Authors:** Ji-Hye Yang, Hye Jin Yang, Chulhwan Choi, Chul-Ho Bum

**Affiliations:** 1 Department of Physical Education, Graduate School, Kyung Hee University, Seocheon-dong 1, Giheung-gu, Yongin-si 17104, Republic of Korea; didwlgp9@khu.ac.kr (J.-H.Y.); y0108577@khu.ac.kr (H.J.Y.); 2 Department of Physical Education, Gachon University, 1342 Seongnamdaero, Sujeong-gu, Seongnam-si 13120, Republic of Korea; 3 Department of Golf Industry, College of Physical Education, Kyung Hee University, Seocheon-dong 1, Giheung-gu, Yongin-si 17104, Republic of Korea

**Keywords:** athletic performance, Big Five model, meta-analysis

## Abstract

Academic interest in athletic performance is ongoing. To examine the correlation between athletic performance and athletes’ personality types, data extraction in line with the Preferred Reporting Items for Systematic Reviews and Meta-Analyses (PRISMA) guidelines was completed in October 2021, and a meta-analysis was performed using 180 data from 18 selected papers using the “meta” package version 4.8-4 of R Studio 3.3.3. As a result, these selected studies proved to have reliable quality in proceeding with this study via quality assessment. The overall effect of personality on athletic performance (AP) was ESr = 0.124, *p* < 0.01. Furthermore, only conscientiousness (ESr = 0.178, *p* < 0.001) and extroversion (ESr = 0.145, *p* < 0.01), among the five personality types, showed statistically significant results, and these two personality types had a positive correlation with performance. In the publication bias test, this study found that (a) agreeableness had a publication bias; but, with an additional test using trim-and-fill, (b) the effect was not significant enough to be considered. In addition, the analysis of the moderating effects was conducted in four aspects, and all moderating effect analyses showed statistically significant differences between the groups, demonstrating the heterogeneity of this study. Therefore, this study found a significant relationship between personality and athletic performance and showed the importance of conscientiousness and extroversion.

## 1. Introduction

Athletic performance (AP) is directly related to athletes’ records and prize money, and academia is aware of the importance of their performance [1]. Factors affecting the AP of athletes in modern society include training [2,3], nutrition [4,5], genetics [6,7], and psychology [8,9,10], and numerous sports field studies (for example [11,12,13]) have been conducted on various factors. Although the proportion is small compared to that of other factors [14], several studies have verified AP based on the personality type of athletes. For example, Han et al.’s [15] results of the study suggested that a group that had low performance showed higher anxiety levels than the other groups that had high performance. Moreover, Piedmont et al. [16] revealed that neuroticism negatively affected game performance.

Costa and McCrae [17] (p. 408) define personality as “the individual’s characteristic styles of thought, feeling, and behavior.” It can be changed by various factors, such as environmental factors [18] or family identities [19]. In Korea, personality-type test results are often shared with others and used as a means of self-analysis. Research on personality types has been primarily conducted from a social perspective [20,21]; however, according to Wolframm et al. [22], personality affects the thoughts and actions of an individual. Therefore, in sports, Calleja-González et al. [23] noted that AP comprises diverse factors such as health, skills, cognition, and personality. Particularly, Raglin’s [24] study found differences in mental health between successful and non-successful athletes via measuring their personalities. Personality is heavily influenced by physical, psychological, and emotional factors, and the personality traits of athletes can be regarded as one of the key factors [25].

Based on budding research that transcended the simple analyses presented in earlier studies on athletes’ personality type ratio [26,27], the current study aimed to prove the significant relationship between personality type and the performance of athletes. For example, Allen et al. [28] stated that athletes’ personality types differed according to their performance, sex, and whether they performed individual or team sports. Kirkcaldy [29] noted that male strikers had a more aggressive and dominant personality than defenders and midfielders, and female strikers had a less extroverted personality and higher neuroticism than other players on the team. However, these results are mixed and inconsistent across various types of sports and research methods because each sport type requires different factors, such as the power of action, teamwork, or calmness.

Personality types can be measured using various tools. Representative tools include the Myers–Briggs Type Indicator (MBTI) [30] and Cattell’s 16 personality factors [31], both of which divide personality into 16 types, and the Big Five [32], which divides personality into five types. In particular, this tool is associated with the Big Five model, which evolved from the detailed personality types by Cattell [31] in the early years to the NEO Personality Inventory (NEO-PI) by Costa and McCrae [32], which is currently the most basic and widely used model [33]. There are five subfactors of the Big Five (Neuroticism, Extroversion, Openness, Agreeableness, and Conscientiousness), and researchers have continued to conduct studies related to personalities from the mid- to late 1900s (for example [34,35]).

According to Piepiora [36], neuroticism refers to the concerned and pessimistic thinking of athletes, such as anxiety, stress, and fear; extroversion refers to sociability with others, extroverted thinking, and creating likeability; openness refers to a lack of aversion to new experiences and high curiosity; agreeableness refers to maintaining cooperative interpersonal relationships, rather than competitive ones; and conscientiousness refers to efforts to achieve their goals in terms of achievement. However, according to Laborde et al. [37], determining the most negative or positive personality types according to the characteristics of each sport is not an easy task. Therefore, studies that have measured the personality types suitable for performing high-risk sports [38], endurance sports [26], and individual or team sports [39] can be said to have significant value.

Furthermore, personality factors that significantly affect AP differ according to the characteristics of each sport. According to Shrivastava et al. [40], athletes’ personalities contribute to high-level performance. In addition, Saale-Prasad [41] noted a significant positive correlation for only agreeableness among the five Big Five personality types, whereas Khan et al. [42] found significant positive and negative results for all five types. As such, this study was conducted to determine the positive and negative effects on player performance via the effect size of five personality factors.

This meta-analysis emphasizes the importance of moderating effects. This is because moderating analysis is commonly used to interpret meta-analyses heterogeneously [43] and helps to prove the statistical power of a meta-analysis [44]. For example, Aulisi et al. [45] not only focused on the role of gender as a moderator, but also Fila et al.’s [46] study proved a significant difference in the moderator effects of gender, and Tipton et al. [47] considered diverse factors such as participant groups or environmental aspects to measure heterogeneity because it is expected that there are some differences in each moderator’s effects. Furthermore, most studies that investigate personality and performance include participants as common persons or students; therefore, it is a deniable fact that this type of study is scarce. Consequently, this study aimed to provide useful data for improving athletes’ performance via integrating the total value with a meta-analysis and moderating analysis and deriving an average result.

### Research Question

The research questions are as follows:

1. What are the effect sizes by personality type? (Neuroticism, Extroversion, Openness, Agreeableness, and Conscientiousness);
2. What are the effect sizes by sport type? (individual, team, and individual or team sports);
3. What are the effect sizes by sex? (male only and both male and female);
4. What are the effect sizes by publication? (academic journal and dissertation);
5. What are the effect sizes by performance measurement methods? (actual and perceived performance).

## 2. Materials and Methods

The guidelines of Preferred Reporting Items for Systematic Reviews and Meta-Analyses (PRISMA) were followed to determine the appropriateness of the literature examined in this study [48]. Moreover, according to Saaiq and Ashraf [49], PICOS is an essential question for researchers to write a systematic and satisfactory paper that consists of four questions: P (population; participants of study), I (intervention; intervention methods and programs), C (comparison; comparative group), and O and S (outcome and study). Thus, this study followed the PICOS questions: (1) adolescent and adult athletes (P), (2) Big Five personalities (I), (3) five types of personality groups (C), and (4) the effects of individual personality on AP (O and S).

### 2.1. Inclusion/Exclusion Criteria

The criteria for non-inclusion in this study are (1) studies that were unrelated to sports, (2) studies not involving professional or elite athletes, (3) studies that did not consider athlete performance and athletes’ personality types as variables, (4) studies that were not full text, and (5) studies that were not written in Korean or English. Conversely, the inclusion criteria for this study were either not mentioned in the non-inclusion criteria or explicitly focused on studies deemed suitable for the research.

### 2.2. Search Strategy

The literature search was completed in October 2021 and a wide spectrum of keywords and diverse databases were used for the systematic review. Moreover, the literature search and paper selection were completed after discussions with all authors. Eight databases were used: Google Scholar, ProQuest, PubMed, Scopus, RISS, KISS, Kyobo Scholar, and DBpia.

To find suitable studies, the authors searched for words related to the topic of this study, gradually added more search words, and designated the search year range until the recent 1900s. Comprehensive searches included various keywords such as sports, athletes, personality, and performance. Synonyms for “personality”, “Big Five”, “MBTI”, and “character” were used, and the word “athletic” was used as a synonym for “athletes”. Therefore, a total of eight keywords were used for the search. In detail, the search sentence searched by title and abstract: (a) “Big Five” OR “MBTI” OR “personality” AND “athletic” AND “performance” AND “sports”; (b) “Big Five” OR “MBTI” OR “personality” OR “character” AND “athletic” AND “performance” AND “sports”; (c) “Big Five” OR “MBTI” OR “personality” OR “character” AND “athletic” OR “athletes” AND “performance” AND “sports”.

Gray literature includes theses and dissertations, conference papers, research reports, committee reports, government reports, and so on [50]. According to Conn et al. [51], a meta-analysis that excluded gray literature was not suitable for stating the exact effect size; therefore, this study highlighted the importance of gray literature. Moreover, recent meta-analyses (for example [52,53]) have included gray literature to show clear results; therefore, this study searched not only common articles but also gray literature, such as dissertations.

### 2.3. Data Items

The extracted data were classified based on all sports (e.g., judo, soccer, basketball, and handball) and the type of sports (individual, team, and individual/team sports). Data were also classified by the athlete’s sex (e.g., only males, only females, both males and females), personality tool used (e.g., Big Five Scale), actual athletic performance tool used (e.g., athletes’ performance discerned by the coach, actual game performance, and achievements), characteristics of comparison or control groups (e.g., excellent/non-excellent groups and champion/general athlete groups that can be used to compare performance), and publication type of the research (academic journal or dissertation).

### 2.4. Quality Assessment

The quality of the papers was examined and the authors agreed to all assessments of methodological quality. Moreover, it was assessed using Cicolini et al.’s [54] Quality Assessment and Validity Tool for Correlational Studies, and the assessment sections are divided into design, sample, measurement, and statistical analysis. The items related to the dependent variable were measured based on the reliability of the AP variable for each study. 

### 2.5. Data Analysis

Data were processed with the “meta” package version 4.8-4 of the R Studio 3.3.3. Moreover, Pearson’s correlation (*r* value) was used in this study; when the selected papers did not have an *r* value and only had a *t* value or mean value, they were all changed to *r* values for use in this study. The *t* and mean values were the values for two groups that could represent AP (e.g., excellent/non-excellent groups, athletes who won championships, and athletes who never won championships). Furthermore, the derived data were analyzed using Fisher’s *Z* test to convert the *r* values into a standardized random effect size, and forest and funnel plots were used to show the visual results.

Moderator analysis, performed to verify heterogeneity, was a categorical variable; thus, a meta-ANOVA was performed. Publishing bias occurs because of the lack of appropriate research for the topic of meta-analysis, and the results of each study cannot be applied [55]. Therefore, for the publication bias test, to determine the asymmetry of the data selected for this study, a funnel plot was used to determine whether the visual effect size was symmetrical. In addition, statistical analyses, Egger’s regression tests, and trim-and-fill tests were performed.

## 3. Results

### 3.1. Literature Search

**Figure 1:**
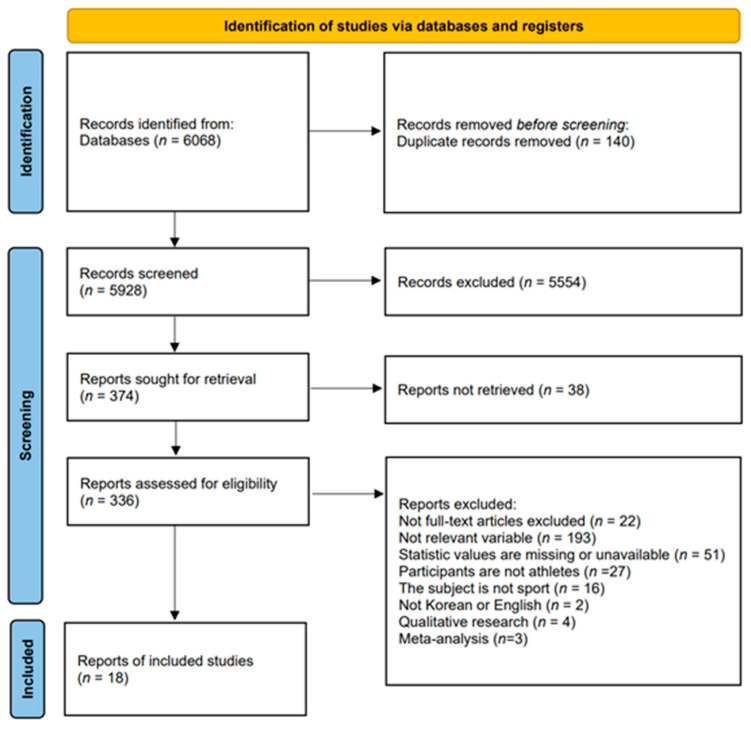
PRISMA flow chart of the literature analyzed in this study.

Eight databases—Google Scholar, ProQuest, PubMed, Scopus, RISS, KISS, Kyobo Scholar, and DBpia—were used to search for 6068 papers. Duplicate papers (*n* = 140) were excluded, and after reviewing the titles and abstracts, the remaining papers (*n* = 374) were selected for an in-depth review. Details are presented in the PRISMA flow chart (Figure 1). A meta-analysis was performed on 18 studies.

### 3.2. Study Characteristics and Results of Quality Assessment

Table 1 summarizes the selected research articles. Eighteen papers were selected for this study and a meta-analysis was performed on 180 datasets derived from 4,101 athletes. The data for this study were based on NEO-PI subfactors, which were derived from the larger scope of the Big Five. The Big Five has five subfactors (Neuroticism, Extroversion, Openness, Agreeableness, and Conscientiousness). The detailed selection criteria for papers related to the moderating effect analysis are presented in Table 2. In the case of the result of quality assessment, eight papers were classified as “high (10–14 points),” ten papers were classified as “medium (5–9 points),” and none were classified as “low (0–4 points).” Therefore, it was concluded that there were no problems in proceeding with this study. Further details are presented in Table 3.

### 3.3. Overall Effects of Personality on AP and Heterogeneity

The data values (*k* = 180) of the 18 selected papers were converted using Fisher’s *Z* value and meta-analyzed using the random-effects model to derive the average effect size of the third decimal digit. The overall ESr was 0.073 (95% confidence interval [CI]: 0.021–0.124; *p* < 0.01). Moreover, in the result of each five personality, ESr was −0.083 (95% CI −0.269; 0.104, *p* = 0.3) for neuroticism (N) (*k* = 33), −0.003 (95% CI-0.066; 0.060, *p* = 0.9) for agreeableness (A) (*k* = 30), 0.035 (95% CI −0.072; 0.142, *p* = 0.5) for openness (O) (*k* = 30), 0.179 for conscientiousness (C) (*k* = 53; 95% CI 0.085; 0.273, *p* < 0.001), and 0.146 (95% CI 0.047; −0.246, *p* < 0.01) for extroversion (E) (*k* = 34). Only C (*p* < 0.001) and E (*p* < 0.01) were statistically significant, with effect sizes smaller than the median of Cohen’s [73] effect size criteria. This means that conscientiousness and extroversion in various sports help athletes achieve higher performance levels. Regarding the heterogeneity of each group, groups N ($I^{2}$ = 97%), A ($I^{2}$ = 78%), O ($I^{2}$ = 92%), C ($I^{2}$ = 93%), and E ($I^{2}$ = 93%) had the highest values.

**Figure 2:**
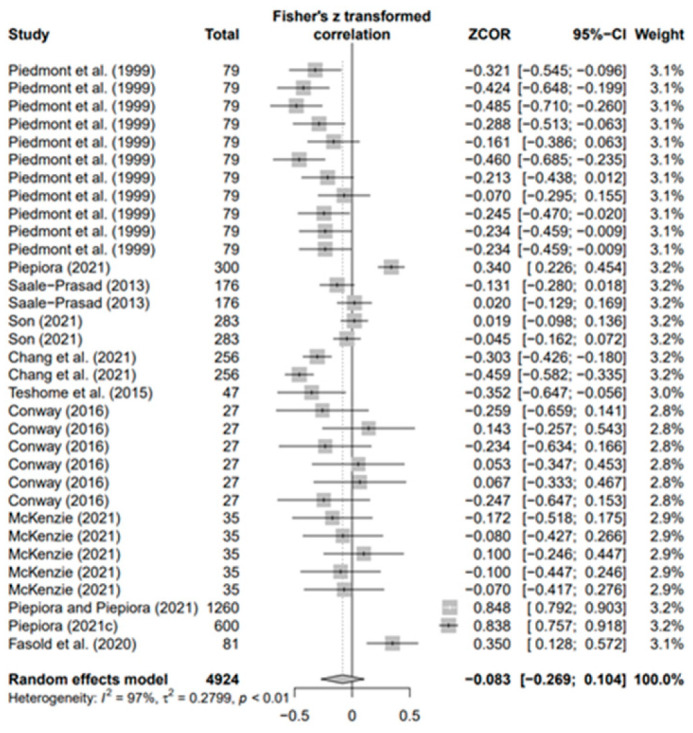
Forest plot of N [16,36,41,56,57,62,63,65,70,71,72].

**Figure 3:**
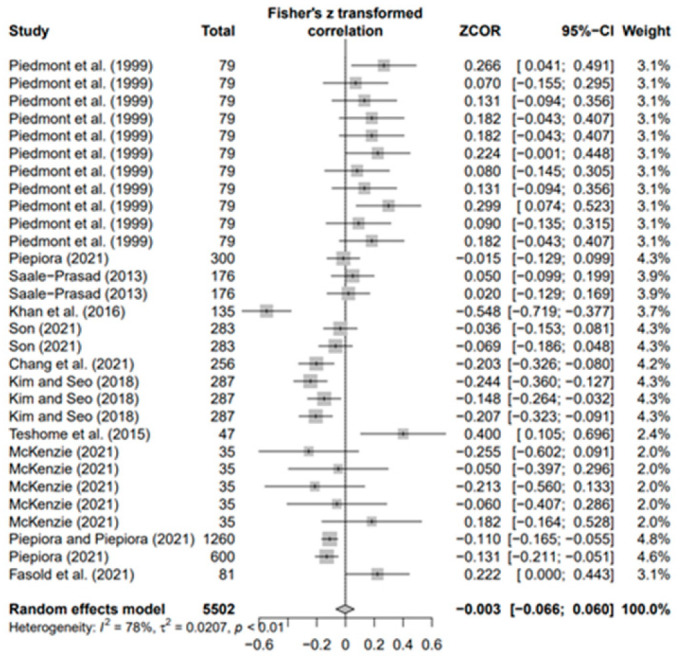
Forest plot of A [16,36,41,42,56,57,58,62,65,70,71,72].

**Figure 4:**
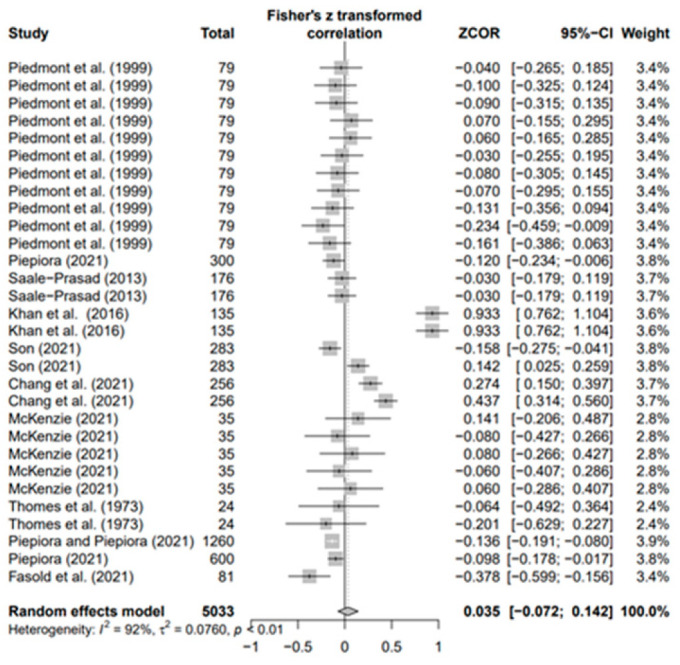
Forest plot of O [16,36,41,42,56,57,65,69,70,71,72].

**Figure 5:**
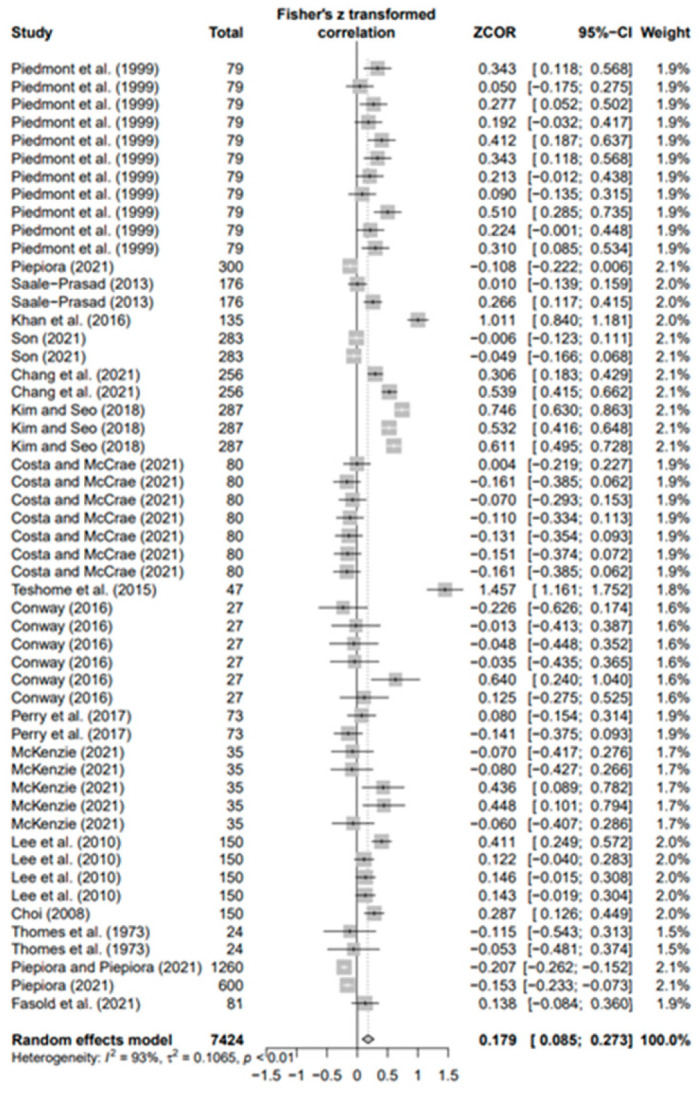
Forest plot of C [16,36,41,42,56,57,58,59,62,63,64,65,66,68,69,70,71,72].

**Figure 6:**
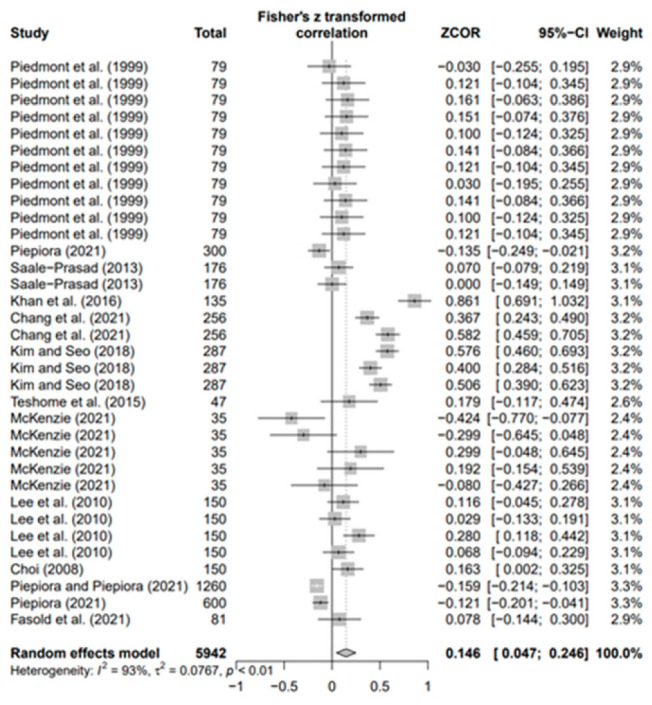
Forest plot of E [16,36,41,42,57,58,62,65,66,68,70,71,72].

**Figure 7:**
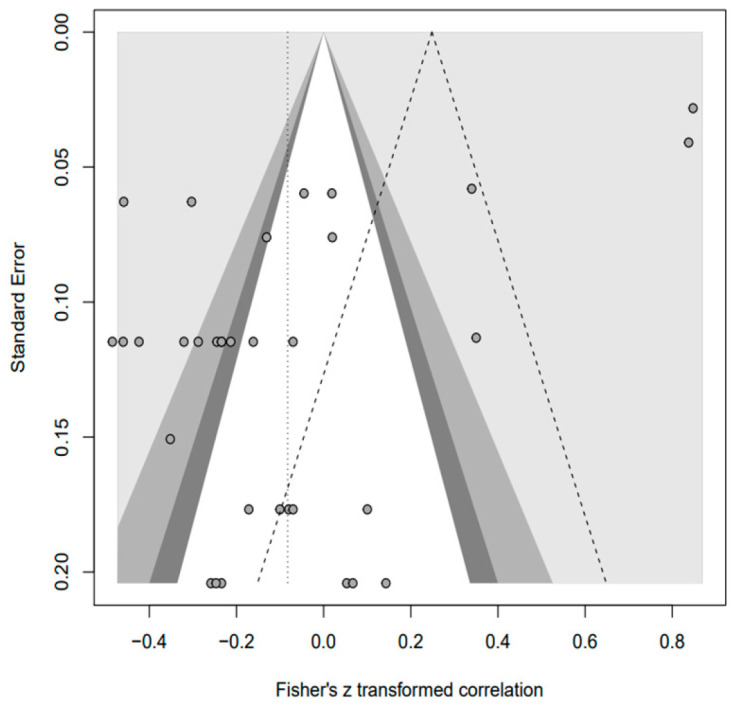
Funnel plot of N.

**Figure 8:**
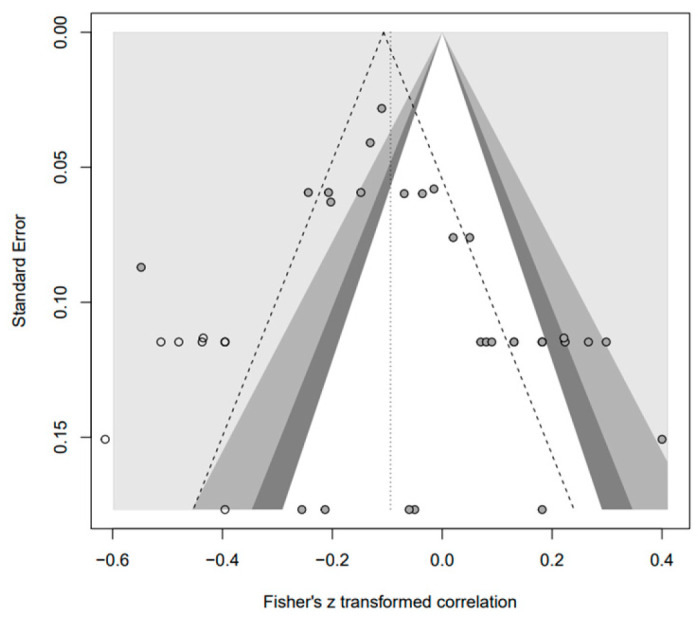
Funnel plot of A.

**Figure 9:**
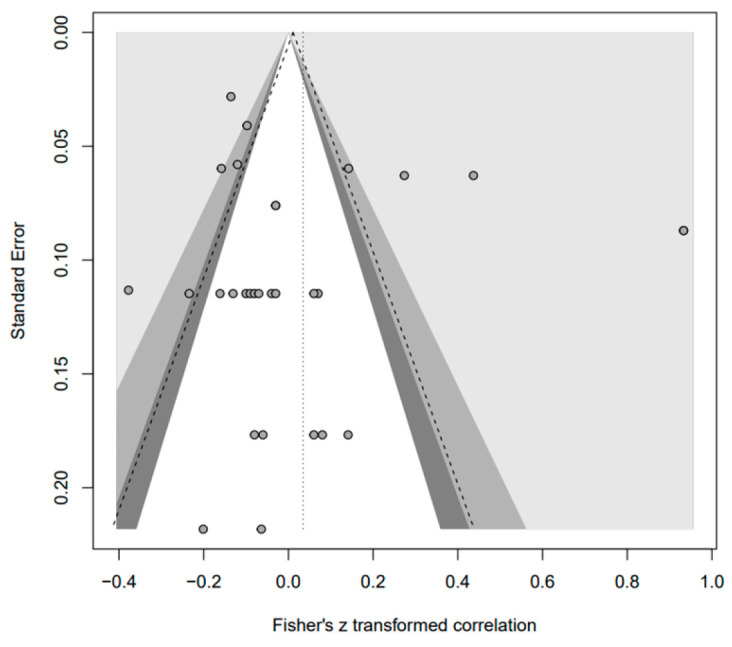
Funnel plot of O.

**Figure 10:**
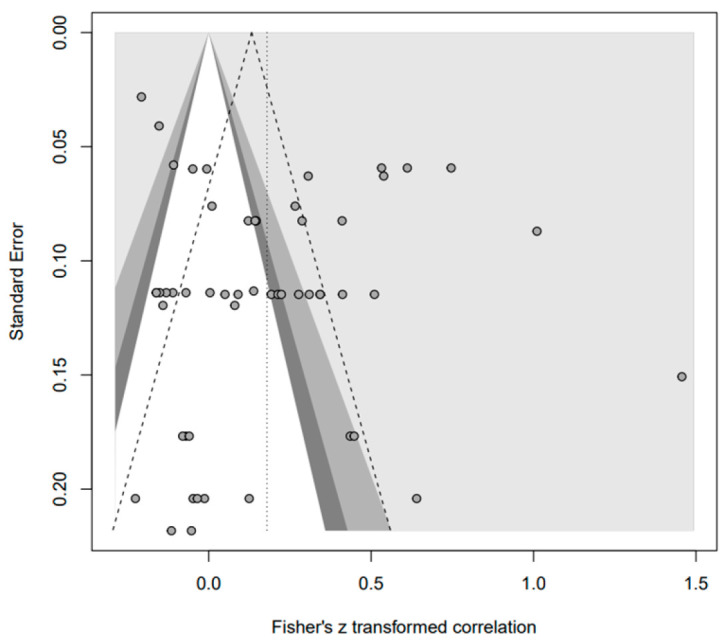
Funnel plot of C.

**Figure 11:**
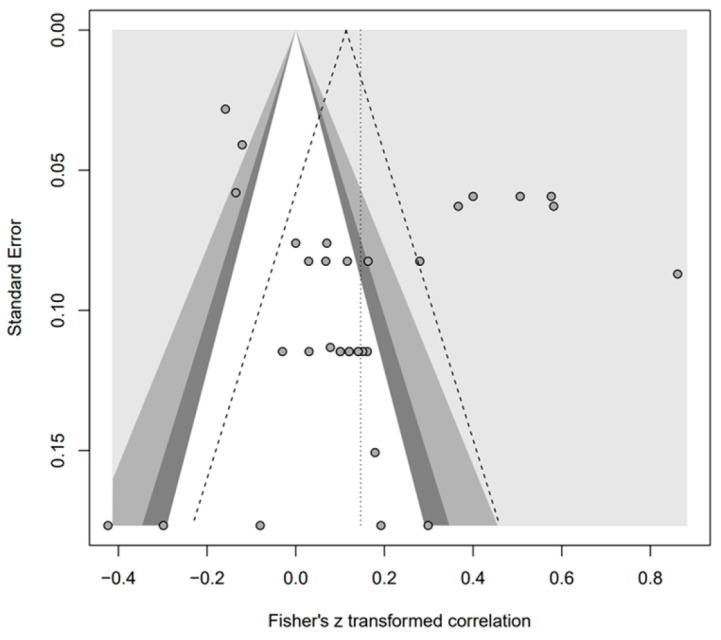
Funnel plot of E.

In addition, the publication bias test was primarily performed using a funnel plot; however, as it was not possible to demonstrate a clear visual publication bias, Egger’s regression test was conducted to verify it via statistical analysis. We found publication bias among the five groups in the data from group A only. However, in the trim-and-fill test, an additional test showed a statistically significant result (*p* < 0.01) after adding data from nine studies; hence, it was determined that this publication bias did not significantly affect the study results. The details of all groups are provided in Figure 2, Figure 3, Figure 4, Figure 5, Figure 6, Figure 7, Figure 8, Figure 9, Figure 10 and Figure 11 and Table 4.

### 3.4. Results of Moderator Analyses

This study found that the heterogeneity of the five groups was quite high ($I^{2}$ = 78–97%). To verify this, the four moderating effects were analyzed using a meta-ANOVA. The moderator analysis of the participants’ sports type was conducted in the same way as the initial data (*k* = 180), and among the five groups, only A and O showed differences. In group A, the amount of data for each subgroup was as follows: individual sports (*k* = 4, weight = 16.8%), individual/team sports (*k* = 12, weight = 39.7%), and team sports (*k* = 14, weight = 43.5%); the difference between the groups was $Q_{B}$ = 35.56, df = 2 (*p* < 0.001). In addition, the subgroup data of O were as follows: individual sports (*k* = 5, weight = 18.5%), individual/team sports (*k* = 10, weight = 32.6%), and team sports (*k* = 15, weight = 48.9%). The difference between the groups was $Q_{B}$ = 8.36, df = 2 (*p* < 0.05), indicating the heterogeneity of the two groups.

When analyzing the moderating effect by gender, there were data on female subjects only in Piedmont et al. [16]; however, as the amount of data was not small (*k* = 11), this study was not excluded. The amount of data in each subgroup of A that was statistically significant was male (*k* = 4, weight = 14.6%), female (*k* = 11, weight = 33.7%), and male and female (*k* = 15, weight = 51.7%). The $Q_{B}$ indicating differences between groups was 27.88 (df = 2, *p* < 0.001). The data for each subgroup of O were male (*k* = 5, weight = 15.9%), female (*k* = 11, weight = 36.9%) and male and female (*k* = 14, weight = 47.2%), and $Q_{B}$ was 8.50 (df = 2, *p* < 0.05), so there was heterogeneity in the two groups, as with sports type.

An analysis of the moderating effect of publication type was conducted using data from journal papers (*n* = 12) and dissertations (*n* = 6). There was a difference between the groups only in group C of the five groups. The amount of data for each subgroup of C was as follows: academic journal (*k* = 30, weight = 58.6%) and dissertation (*k* = 23, weight = 41.4%). The difference between groups, $Q_{B}$, was 6.68 (df = 1, *p* < 0.01), which is statistically significant and can constitute evidence for heterogeneity.

The methods for performance measurement were divided into measures of perceived performance, such as questionnaire surveys or coach ratings; measures of performance via athletes’ actual match statistics; frequencies of gold medal acquisition; and rankings. The analysis of moderating N showed significant results, and the amount of data in each subgroup was perceived performance (*k* = 10, weight = 29.5%) and actual performance (*k* = 23, weight = 70.5%). The differences between the groups were also statistically significant ($Q_{B}$ = 20.90, df = 1, *p* < 0.001), which could explain the heterogeneity of the data in this group. The details of the overall results for the moderator effects are presented in Table 5, Table 6, Table 7 and Table 8.

## 4. Discussion

This study aimed to determine the relationship between the personality types of elite sports athletes and their AP via conducting a meta-analysis. Thus, this study found that two personality traits (conscientiousness and extroversion) among the Big Five were related to and correlated with AP. However, some scholars have noted that personality types in sports contexts may not be reliable because of biased research results from a small number of people [74]. However, recent studies have examined the effect of sports participation on personality [75,76] and presented practical data for enhancing social skills. Moreover, recent studies have derived different personality types [77,78] according to the characteristics of the sport (e.g., high risk, individual sports, and team sports), thus making it possible to predict participants’ sports selection. Owing to these recent studies, the reliability of personality test results has increased.

Although the number of studies that completely matched the purpose of the current study was scant, studies that focused on personality types, such as those only on personality types [26] or persons who were not athletes, provided great insight [79]. The need for the current study was apparent from the results of the meta-analysis. Studies on elite athletes’ personality types have primarily focused on physical activity [80,81,82], while some studies have focused on the relationship between personality and intended practice [83]. However, few meta-analyses have been performed with the variables used in this study because there were relatively fewer athletes than office workers and students; thus, the necessity of this study is palpable. The results of this study demonstrate that the correlation between personality traits and the performance of athletes in various sports was statistically significant.

To elaborate on the meta-analysis results, Weibel’s [84] research stated that extroversion was also important for immersion, which significantly affects performance. Rosander et al. [85] showed that, among the Big Five factors, only conscientiousness and extroversion had a positive effect even in general school sports classes, which are not competitive sports competitions. Nia and Besharat [39] noted that athletes have higher levels of conscientiousness and extroversion than non-athletes, signifying that these personality factors are highly correlated with athletic ability. Khan et al. [42] stated that extroversion and conscientiousness provide benefits for AP, such as reducing anxiety and maintaining athlete composure. Therefore, combining various data values from the 18 papers selected for this study showed that their results were similar to those of previous studies. 

This study integrated data on all sports categories and types and obtained the same results as previous studies, thereby showing a significant relationship between conscientiousness and extroversion. However, other factors were found to be statistically insignificant. Moreover, moderator analyses showed significant differences between the subgroups of sports type (individual, team, and individual/team sports), gender (only male, only female, both male and female), publication type (academic journals, dissertations), and performance test method type (actual and perceived performance), as seen in recent studies. For example, Wisniewski et al. [86] found differences in the moderator effect of publication type, whereas Bruner et al. [87] found a moderating effect of sports type on study heterogeneity. Thus, the results of this study will be useful in improving the quality of life of elite athletes via presenting data that can help enhance their AP.

In summary, this study found partially significant relationships between AP and five personality traits. These results may vary depending on the situation and characteristics of sports; therefore, it is not possible to affirm what kinds of characteristics are essential. However, the purpose of the meta-analysis was to draw conclusions from previous studies [88], so the results will be useful and basic data to determine which personality traits are overall good for athletes.

### Limitations and Future Directions

A meta-analysis integrates and analyzes various values from different papers on the same subject into a single value. To derive more reliable results, the selection, validity, and quality evaluation of the papers are important. This study was systematically conducted to present the correlation between all personality types and AP and the different influences depending on the subfactors of personality types. However, this study has a few limitations.

This study focused on individuals who participated in a variety of sports. However, it is important to study only the relationship between certain athletes’ personalities and performance. Thus, it is necessary to conduct a detailed follow-up study of certain sports such as ball, team, and individual sports. Moreover, when utilizing statistical values, some had incomplete statistical tables with the r value, that is, correlation data; thus, this study requested the authors for originals. However, this study did not receive any responses; therefore, it was excluded. Studies on college athletes/professional athletes and senior/junior athletes were excluded. This is because most AP depends on the individual level of the participants [89]. Therefore, papers without a clear grade standard were deemed unsuitable.

## 5. Conclusions

This study conducted a systematic review and meta-analysis to investigate the association between personality types and AP. Of the five personality types, only conscientiousness (C) and extroversion (E) had statistically significant positive (+) effects on performance. Although the publication bias test showed biased data for agreeableness (A), the statistical significance was the same even after adding nine additional data points in the trim-and-fill. Thus, we determined that the publication bias was not significant enough to affect the study results. To test heterogeneity, a moderation effect analysis was conducted on four aspects (sport, sex, publication, and performance measurement method). There were significant differences between groups A and O in sports and sex types, between groups C in publication type, and among group N in performance measurement type. These results may constitute evidence of the heterogeneity in this study. The results of this study will be valuable in identifying the influence of personality factors on the performance of athletes in various sports and may be used to help athletes achieve higher performance.

## Figures and Tables

**Table 1:** Summary of all the studies’ characteristics.

Studies	Participant Characteristics	Extracted Value (*r*, *t,* mean)	Personality Questionnaires	Performance Questionnaires
[56] (2021)	South Korean male/female weightlifters (283 people)	Correlation (*r*)	Big Five	AP
[57] (2021)	South Korean elite athletes (256 people)	Correlation (*r*)	International Personality Item Pool: IPIP	AP
[58] (2018)	Athletes in the 16 sports categories listed in the Korea Sports Council (287 people)	Correlation (*r*)	5-factor personality scale used by [59,60], etc.	AP
[61] (2021)	Student athletes above the age of 18 (80 people)	Correlation (*r*)	16 Personality Factor Model	Coach Rating Scale,
[41] (2013)	Athletes in individual and team sports (176 people)	Correlation (*r*)	NEO-PI-3	Percentage of Games Started in a Season
[42] (2016)	Athletes with a championship title (135 people)	Correlation (*r*)	Ten Item Personality Inventory (TIPI)	Coaches’ ratings
[62] (2015)	Soccer players who are members of the Aba Buna & Jimma Kenema soccer team (51 people)	Correlation (*r*)	NEO-PI-R	Ratio of gold medals won to the number of competitions the athlete participated in
[16] (1999)	Female soccer players from 4 universities (79 people)	Correlation (*r*)	Big five	Coach’s ratings
[63] (2016)	Junior hockey team members (27 people)	Correlation (*r*)	Big Five	Coach’s ratings, Performance indices
[64] (2017)	Athletes in individual sports above the age of 18 (73 people)	Correlation (*r*)	Ten Item Personality Inventory (IPIP)	Game Statistic
[65] (2021)	Member of the university basketball, hockey, golf, and track and field teams in Canada (35 people)	Correlation (*r*)	Big five	AP Subjective Rating Scale (APSRS)
[66] (2010)	South Korean high-school swimming athlete (150 people)	*t* value	Personality scale used by [67]	Coach’s ratings
[68] (2008)	South Korean high-school swimming athlete (150 people)	Excellent group (52 athletes)	Personality scale used by [67]	Excellent group standard
[69] (1973)	Male football players from Central Catholic Highschool (24 people)	Non-excellent group (98 athletes)	Cattell’s 16 personality factors	Ranked in the top 3 in a national competition at least once
[70] (2021)	Athletes from various sports categories (1260 people)	*t* value	NEO-FFI	Excellent group standard
[36] (2021)	Polish athletes from various team sports categories (300 people)	Excellent group (52 athletes)	NEO-FFI	Ranked in the top 3 in a national competition at least once
[71] (2021)	Athletes from various individual sports categories (600 people)	Non-excellent group (98 athletes)	NEO-FFI	Coach’s ratings
[72] (2020)	Male handball goal keepers playing in the German league (81 people)	*t* value	BFI-10	Champion standard

**Table 2:** Modulator effect variable data for each study.

Studies	Personality Type (Big Five-NEO)	Athletes’ Sports Type	Gender Type	Publication Type	Performance Test Type
[56] (2021)	Included	Individual sports	Male/Female	Dissertation	Perceived performance
[57] (2021)	Included	Individual/Team sports	Male/Female	Academic journal	Perceived performance
[58] (2018)	Partially Included (Conscientiousness. Agreeableness, Extroversion)	Individual/Team sports	Male/Female	Academic journal	Perceived performance
[59] (2021)	Partially Included (Conscientiousness)	Team sports	Male/Female	Dissertation	Perceived performance
[41] (2013)	Included	Individual/Team sports	Male/Female	Dissertation	Perceived performance
[42] (2016)	Partially Included (Extroversion, Conscientiousness, Openness, Agreeableness)	Individual sports	Male/Female	Academic journal	Actual performance
[62] (2015)	Included	Team sports	Male	Academic journal	Perceived performance
[16] (1999)	Included	Team sports	Female	Academic journal	Perceived performance
[63] (2016)	Partially Included (Conscientiousness, Neuroticism)	Team sports	Male	Dissertation	Actual performance
[64] (2017)	Partially Included (Conscientiousness)	Individual sports	Male/Female	Academic journal	Perceived performance
[65] (2021)	Included	Individual/Team sports	Male/Female	Dissertation	Perceived performance
[66] (2010)	Partially Included (Conscientiousness, Extroversion)	Individual sports	Male/Female	Academic journal	Actual performance
[68] (2008)	Partially Included (Conscientiousness, Extroversion)	Individual sports	Male/Female	Dissertation	Actual performance
[69] (1973)	Included	Team sports	Male	Academic journal	Perceived performance
[70] (2021)	Included	Individual/Team sports	Male	Academic journal	Actual performance
[36] (2021)	Included	Team sports	Male	Academic journal	Actual performance
[71] (2021)	Included	Individual sports	Male/Female	Academic journal	Actual performance
[72] (2020)	Included	Team sports	Male	Academic journal	Actual performance

**Table 3:** Quality assessment and validity of the correlational studies.

Studies	Q1	Q2-1	Q2-2	Q2-2	Q2-4	Q2-5	Q3-1	Q3-2	Q4-1	Q4-2 (2 Point)	Q4-3	Q5-1	Q5-2	Outcome
[56]	Y	N	Y	Y	Y	Y	Y	Y	Y	Y	Y	Y	Y	13 (H)
[57]	Y	N	Y	N/A	Y	Y	Y	Y	Y	Y	Y	Y	Y	12 (H)
[58]	Y	N	Y	N	Y	Y	Y	Y	Y	Y	Y	Y	Y	12 (H)
[59]	Y	N	Y	N	Y	N/A	Y	Y	Y	Y	Y	Y	Y	11 (H)
[41]	Y	N	Y	Y	Y	N/A	Y	Y	N	N	Y	Y	Y	9 (M)
[42]	Y	Y	Y	N	Y	Y	Y	Y	N	N	Y	Y	Y	10 (H)
[62]	Y	N	Y	N	Y	N/A	Y	Y	Y	Y	Y	Y	Y	12 (H)
[16]	Y	N	Y	Y	Y	N/A	Y	Y	Y	Y	Y	Y	Y	12 (H)
[63]	Y	N	Y	Y	Y	N/A	Y	Y	N	N	Y	Y	Y	9 (M)
[64]	Y	N	Y	Y	Y	N/A	Y	Y	Y	Y	Y	Y	Y	12 (H)
[65]	Y	N	Y	Y	Y	N/A	Y	Y	N	N	Y	Y	Y	9 (M)
[66]	Y	N	Y	N	Y	Y	Y	Y	N/A	N/A	Y	N	Y	8 (M)
[68]	Y	N	Y	N	Y	Y	Y	Y	N/A	N/A	Y	N	Y	8 (M)
[69]	Y	N	Y	Y	Y	N/A	Y	Y	N/A	N/A	Y	N	Y	8 (M)
[70]	Y	N	Y	N/A	Y	N/A	Y	Y	N/A	N/A	Y	N	Y	7 (M)
[36]	Y	N	Y	N/A	Y	N/A	Y	Y	N/A	N/A	Y	N	Y	7 (M)
[71]	Y	N	Y	N/A	Y	N/A	Y	Y	N/A	N/A	Y	N	Y	7 (M)
[72]	Y	N	Y	Y	Y	N/A	Y	Y	N/A	N/A	Y	N	Y	8 (M)

Note: Y = Yes, N = No, N/A = No Answer, Q1 = Was the study prospective? Q2-1: Was probability sampling used? Q2-2: Was the sample size justified? Q2-3: Was the sample drawn from more than one site? Q2-4: Was anonymity protected? Q2-5: Was the response rate greater than 60%? Q3-1: Was the outcome measured reliably? Q3-2: Was the outcome measured using a valid instrument? Q4-1: Was the dependent variable measured using a valid instrument? Q4-2: If a scale was used for measuring the dependent variable, was the internal consistency ≥ 0.70? Q4-3: Was a theoretical framework used for the guidance? Q5-1: If multiple outcomes are studied, are the correlations analyzed? Q5-2: Were the outliers managed? H = High, M = Medium.

**Table 4:** Meta-analysis results of personality types and AP.

	*k*	ESr	Confidence Interval	$I^{2}$	Egger’s (t)	Egger’s (df)	Egger’s (*p*)	Trim-and-Fill
1->6	*k* = 33	−0.083	−0.269; 0.104	97% ***	−5.037	31	1.927	-
2->6	*k* = 30	−0.003	−0.066; 0.060	78% ***	3.032	28	0.005 **	9 research papers added **
3->6	*k* = 30	0.035	−0.073; 0.142	92% ***	1.857	49	0.069	-
4->6	*k* = 53	0.179 ***	0.085; 0.273	93% ***	0.811	28	0.423	-
5->6	*k* = 34	0.146 **	0.047; 0.246	93% ***	1.356	32	0.184	-

1: Neuroticism, 2: Agreeableness, 3: Openness, 4: Conscientiousness, 5: Extroversion, 6: Athletic performance. *p* < 0.01 **, *p* < 0.001 ***.

**Table 5:** Moderator analysis results of types of sports.

	*k*	ESr	$I^{2}$	$Q_{B}$ (df)	*p* Value
1->6	3	0.272	99% **	1.87 (2)	*p* = 0.3
1->7	20	−0.166	84% **		
1->8	10	−0.033	99% **		
2->6	4	−0.184	89% **	35.56 (2)	*p* < 0.001 ***
2->7	14	0.147	16%		
2->8	12	−0.122	47% *		
3->6	5	0.345	98% **	8.3 (2)	*p* < 0.05 *
3->7	15	−0.106	0%		
3->8	10	0.072	90% **		
4->6	11	0.168	95% **	1.35 (2)	*p* = 0.5
4->7	29	0.138	84% **		
4->8	13	0.277	97% **		
5->6	7	0.196	95% **	0.79 (2)	*p* = 0.6
5->7	14	0.172	19%		
5->8	13	0.065	96% **		

1: Neuroticism; 2: Agreeableness; 3: Openness; 4: Conscientiousness; 5: Extroversion; 6: Only individual sports; 7: Only team sports; 8: Both individual sports and team sport. * *p* < 0.05, ** *p* < 0.01, *** *p* < 0.001.

**Table 6:** Moderator analysis results of gender.

	*k*	ESr	$I^{2}$	$Q_{B}$ (df)	*p* Value
1->6	12	−0.030	98% **	3.81 (2)	*p* = 0.1
1->7	10	0.089	98% **		
1->8	11	−0.285	20%		
2->6	15	−0.137	70% **	27.88 (2)	*p* < 0.001 ***
2->7	4	0.047	84% **		
2->8	11	0.165	0%		
3->6	14	0.191	95% **	8.5 (2)	*p* < 0.05 *
3->7	5	−0.181	15%		
3->8	11	−0.073	0%		
4->6	30	0.164	94% **	1.11 (2)	*p* = 0.5
4->7	12	0.130	92% **		
4->8	11	0.269	28%		
5->6	19	0.207	94% **	3.2 (2)	*p* = 0.2
5->7	4	−0.027	65% *		
5->8	11	0.105	0%		

1: Neuroticism; 2: Agreeableness; 3: Openness; 4: Conscientiousness; 5: Extroversion; 6: Only male; 7: Only female; 8: Both male and female. * *p* < 0.05, ** *p* < 0.01, *** *p* < 0.001.

**Table 7:** Moderator analysis results of publishing type.

	*k*	ESr	$I^{2}$	$Q_{B}$ (df)	*p* Value
1->6	18	−0.100	98% **	0.04 (1)	*p* = 0.8
1->7	15	−0.040	0%		
2->6	21	0.014	83% **	0.39 (1)	*p* = 0.5
2->7	9	−0.028	0%		
3->6	21	0.045	94% **	0.12 (1)	*p* = 0.7
3->7	9	−0.005	43%		
4->6	30	0.283	96% **	6.68 (1)	*p* < 0.01 **
4->7	23	0.026	61% **		
5->6	26	0.186	94% **	2.24 (1)	*p* = 0.1
5->7	8	0.014	58% *		

1: Neuroticism; 2: Agreeableness; 3: Openness; 4: Conscientiousness; 5: Extroversion; 6: Academic journal; 7: Dissertation. * *p* < 0.05, ** *p* < 0.01.

**Table 8:** Moderator analysis results of performance test method type.

	*k*	ESr	$I^{2}$	$Q_{B}$	*p* value
1->6	10	−0.208	70% **	20.09 (1)	*p* < 0.001 ***
1->7	23	0.275	95% **		
2->6	25	0.028	72% **	3.5 (1)	*p* = 0.06
2->7	5	−0.123	89% **		
3->6	24	−0.008	74% **	1.92 (1)	*p* = 0.1
3->7	6	0.184	98% **		
4->6	38	0.198	90% **	0.44 (1)	*p* = 0.5
4->7	15	0.132	94% **		
5->6	24	0.166	85% **	0.33 (1)	*p* = 0.5
5->7	10	0.111	95% **		

1: Neuroticism; 2: Agreeableness; 3: Openness; 4: Conscientiousness; 5: Extroversion; 6: Perceived athletic performance; 7: Real athletic performance. ** *p* < 0.01, *** *p* < 0.001.
